# IDH-1 deficiency induces growth defects and metabolic alterations in GSPD-1-deficient *Caenorhabditis elegans*

**DOI:** 10.1007/s00109-018-01740-2

**Published:** 2019-01-19

**Authors:** Hung-Chi Yang, Hsiang Yu, You-Cheng Liu, Tzu-Ling Chen, Arnold Stern, Szecheng J. Lo, Daniel Tsun-Yee Chiu

**Affiliations:** 1 Department of Medical Laboratory Science and Biotechnology, Yuanpei University of Medical Technology, Hsinchu, Taiwan; 2 Department of Medical Biotechnology and Laboratory Sciences, College of Medicine, Chang Gung University, Taoyuan, Taiwan; 3 Graduate Institute of Biomedical Sciences, College of Medicine, Chang Gung University, Taoyuan, Taiwan; 4 New York University School of Medicine, New York, NY USA; 5 Department of Biomedical Sciences, College of Medicine, Chang Gung University, Taoyuan, Taiwan; 6 Research Center for Chinese Herbal Medicine, College of Human Ecology, Chang Gung University of Science and Technology, Taoyuan, Taiwan; 7 Department of Pediatric Hematology/Oncology, Linkou Chang Gung Memorial Hospital, Taoyuan, Taiwan; 8 Healthy Aging Research Center, Chang Gung University, Taoyuan, Taiwan

**Keywords:** *C. elegans*, GSPD-1, IDH-1, Development, Molting, Metabolomic, Amino acid

## Abstract

NADPH is a reducing equivalent that maintains redox homeostasis and supports reductive biosynthesis. Lack of major NADPH-producing enzymes predisposes cells to growth retardation and demise. It was hypothesized that double deficiency of the NADPH-generating enzymes, GSPD-1 (Glucose-6-phosphate 1-dehydrogenase), a functional homolog of human glucose-6-phosphate dehydrogenase (G6PD), the rate-limiting enzyme of the pentose phosphate pathway, and IDH-1 (isocitrate dehydrogenase-1) affect growth and development in the nematode, *Caenorhabditis elegans* (*C. elegans*). The *idh-1*;*gspd-1*(*RNAi*) double-deficient *C. elegans* model displayed shrinkage of body size, growth retardation, slowed locomotion, and impaired molting. Global metabolomic analysis was employed to address whether or not metabolic pathways were altered by severe NADPH insufficiency by the *idh-1*;*gspd-1*(*RNAi*) double-deficiency. The principal component analysis (PCA) points to a distinct metabolomic profile of *idh-1*;*gspd-1*(*RNAi*) double-deficiency. Further metabolomic analysis revealed that NADPH-dependent and glutamate-dependent amino acid biosynthesis were significantly affected. The reduced pool of amino acids may affect protein synthesis, as indicated by the absence of NAS-37 expression during the molting process. In short, double deficiency of GSPD-1 and IDH-1 causes growth retardation and molting defects, which are, in part, attributed to defective protein synthesis, possibly mediated by altered amino acid biosynthesis and metabolism in *C. elegans*.

**Electronic supplementary material:**

The online version of this article (10.1007/s00109-018-01740-2) contains supplementary material, which is available to authorized users.

## Introduction

Glucose-6-phosphate dehydrogenase (G6PD) is the first and rate-limiting enzyme in the hexose monophosphate shunt (HMS), also known as the pentose phosphate pathway (PPP). The classical biochemical role of G6PD is to catalyze the oxidation of glucose-6-phosphate to 6-phosphogluconolactone and concomitantly produce the reduced form of nicotinamide adenine dinucleotide phosphate (NADPH) for antioxidant defense and reductive biosynthesis [1, 2]. G6PD deficiency is the most common enzymopathy affecting 400 million people in the world. Most G6PD mutations are point mutations causing amino acid substitution and reduced enzyme activity. Classically, G6PD deficiency has been linked to red blood cell disorders as the clinical manifestations. Novel functions of G6PD in cellular physiology have been discovered over recent years [3]. G6PD is required for life as severe deficiency of G6PD in mammals and nematodes is lethal for survival and reproduction [4, 5] and plays an important role in embryogenesis [6, 7].

The biological importance of G6PD is largely attributed to the production of NADPH. The reducing equivalent NADPH serves pivotal roles in cellular biology. It maintains cellular redox homeostasis by regenerating reduced glutathione, which is needed for detoxifying elevated oxidants. NADPH is also required for reductive biosynthesis of building blocks. Intracellular NADPH is supported by parallel pathways that are localized in different cellular compartments [8]. In the cytoplasm, the main source of NADPH is the oxidative branch of the PPP. Alternative NADPH-producing pathways include cytosolic malic enzyme (ME1), cytosolic and mitochondrial isocitrate dehydrogenase (IDH1 and IDH2), transhydrogenase (NNT), and cytosolic and mitochondrial methylene tetrahydrofolate dehydrogenase (MTHFD1 and MTHFD2) [9]. Understanding how compartmentalization of NADPH homeostasis affecting cell development in health and diseases presents a major investigative challenge.

The biochemical role of IDHs is the oxidation of isocitrate to oxalosuccinate followed by the decarboxylation and ultimately the production of alpha-ketoglutarate, which is coupled with the reduction of NADP^+^ to NADPH. In humans, IDH1 and IDH2 are NADP^+^-dependent enzymes, while IDH3 is a NAD^+^-dependent multi-subunit mitochondrial enzyme. Human IDH1 localizes in the cytoplasm and peroxisome and is highly expressed in the liver. Human IDH2, which has the mitochondrial signal peptide at the amino terminus, localizes in mitochondria and is highly expressed in mammalian muscle, heart, and lymphocyte [10]. IDH1 is associated with lipid metabolism and glucose sensing [11, 12], whereas IDH2 regulates oxidative respiration [13]. In *Caenorhabditis elegans* (*C. elegans*), IDH-1 is predicted as a cytosolic enzyme, whereas IDH-2 is predicted as a mitochondrial enzyme [14]. Although the enzymatic activity of IDH1 has been discovered decades ago, the biological function of wild type (WT) IDH1 is still controversial. Compared to WT IDH1, oncogenic IDH1/2 mutants are well studied and received considerable attention. IDH1/2 mutations have been found in glioma, glioblastoma, acute myelogenous leukemia, chondrosarcoma, and enchondroma [15, 16]. Animal study shows that IDH null mice are healthy and fertile at steady state, while the liver displays altered amino acid utilization [17, 18].

How severe NADPH insufficiency, as a consequence of impairment of different NADPH-producing systems, affects metabolic networks and links to pathophysiology of an organism has not been defined. The notion that NADPH is indispensable for life is supported by the fact that knockdown of NADPH-producing enzymes leads to growth arrest and cell demise. G6PD-deficient human foreskin fibroblasts display slow growth and early onset of senescence [19]. A yeast model with co-disruption of major NADPH sources (G6PD and cytosolic IDH) undergoes growth inhibition and loses viability [20]. Whereas loss of G6PD or IDH alone fails to induce a growth defect, a NADPH compensatory mechanism at the cellular level may maintain NADPH homeostasis and warrant normal development. However, the contribution from each NADPH-producing system at the organismal level remains unclear.

Further study of NADPH-producing systems by applying metabolomic technology to *gspd-1* (*G6PD* homolog) and *idh-1* double-deficient *C. elegans* model will help in clarifying the role of redox homeostasis and regulation in growth and development. Metabolomics is a novel platform of systems biology that aims to characterize all small molecule metabolites (metabolome) in various forms of biological samples. It is a powerful tool to most closely reflect phenotypic expression and it acutely pinpoints the perturbations within metabolic networks. Such metabolic disturbances can be attributed to downstream alterations of genomic and proteomic outcomes. Current advances place metabolomics in the armamentarium of cutting-edge strategies to dissect the metabolic networks of human and animal models in health and diseases. The intermediary metabolic network is conserved among eukaryotic organisms. The nematode *C. elegans* has orthologs for most human metabolic enzymes, including G6PD and IDH1 [21]. *C. elegans* is a simple and ideal biological system to model human metabolic disturbances. A number of *C. elegans* studies have taken advantage of different metabolomic approaches, including nuclear magnetic resonance (NMR) spectroscopy, gas/liquid chromatography-coupled mass spectrometry (GC/LC-MS) for analyzing the metabolic pathways in a whole worm [22–28]. Lipidomics has been employed in characterizing the molecular pathway in *gspd-1*(*RNAi*) *C. elegans*. Embryonic lethality occurs through the induction of lipid oxidative damage and activation of lipid-modifying enzymes as identified by lipidomics [6].

In the current study, a global metabolomic platform has been employed for analyzing the metabolome of *idh-1*;*gspd-1*(*RNAi*) double-deficient *C. elegans*. Phenotypic characterization has shown that several developmental impairments are found in this double mutant, including defective molting and reduced growth (small body size, delayed growth, and slowed locomotion). The global metabolomic study has also shown that several amino acid metabolic pathways are altered, most notably in those amino acids requiring NADPH for their synthesis and metabolism, such as the biosynthesis of valine, leucine, isoleucine; phenylalanine, tyrosine, and tryptophan biosynthesis as well as the metabolism of glutamine and glutamate. These findings enhance our understanding of the causal relationship between insufficient NADPH supply, altered amino acid metabolism, and the resulting developmental defects.

## Results

### Growth retardation and molting defect of *idh-1*;*gspd-1*(*RNAi*) double-deficient *C. elegans*

**Fig. 1:**
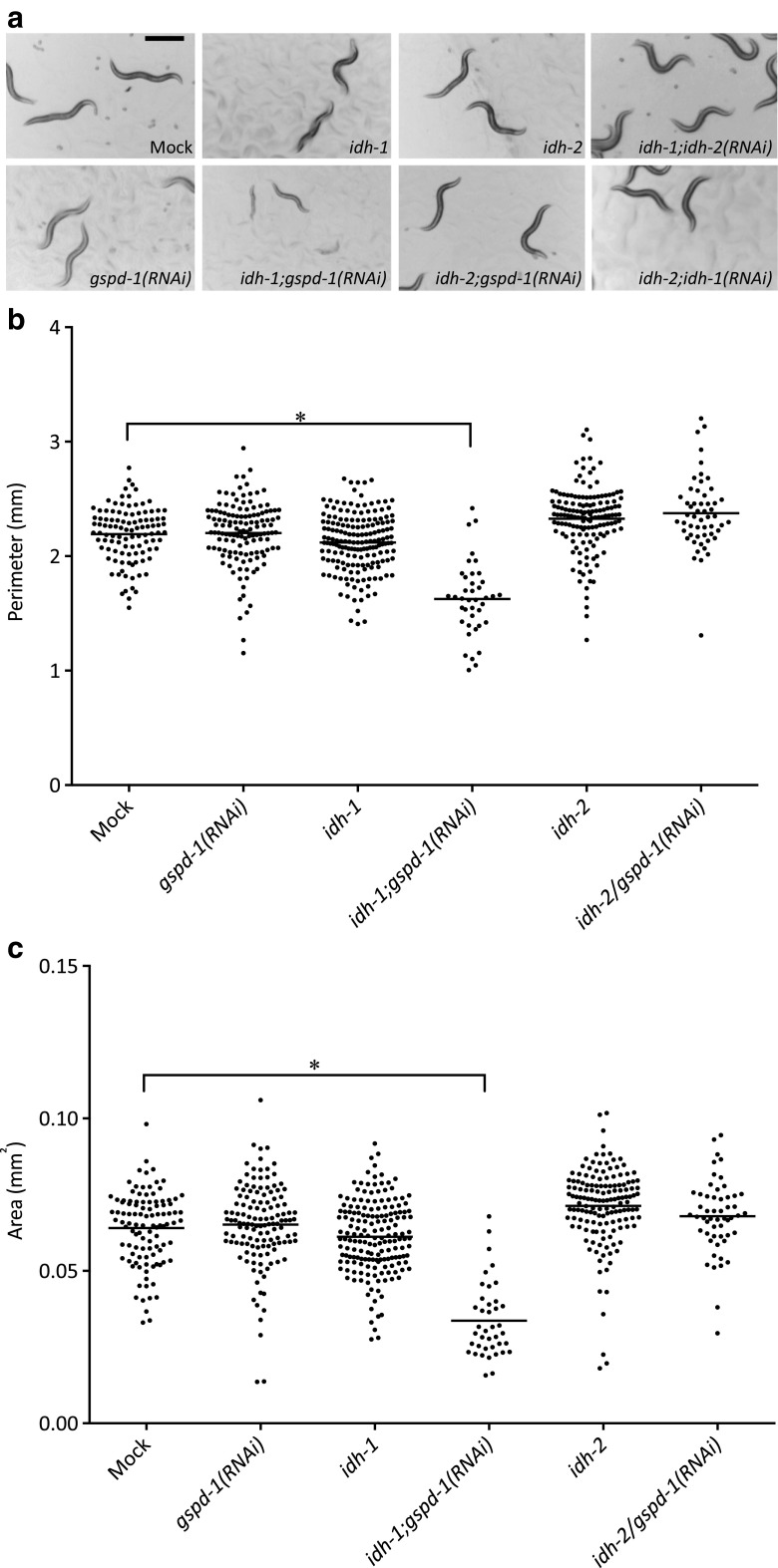
Decreased body size of *idh-1*;*gspd-1*(*RNAi*) double-deficient *C. elegans* compared to mock and other controls. (**a**) The size of *idh-1*;*gspd-1*(*RNAi*) double-deficient *C. elegans* was decreased compared to other *C. elegans* strains at 72 h. Adult *C. elegans* were examined by image analysis software under dissecting microscope. *Idh-1*;*gspd-1*(*RNAi*) double-deficient *C. elegans* showed decreased perimeter (**b**) and area (**c**) compared to other *C. elegans* strains at 72 h. Each dot represented one adult worm. Horizontal line represented the mean of each *C. elegans* strain. The black scale bar represented 0.5 mm (*n* ≥ 40, *P < 0.05)

Mutants of isocitrate dehydrogenases were used to investigate the complementary roles of cytoplasmic NADPH-producing pathways during growth and development. In brief, *gspd-1* RNAi was used in *idh-1* and *idh-2* deletion mutants to generate *idh-1*;*gspd-1*(*RNAi*) and *idh-2*;*gspd-1*(*RNAi*) double-deficient *C. elegans*, respectively (Supplementary Fig. S1). After 72 h, *idh-1*;*gspd-1*(*RNAi*) double-deficient *C. elegans*, compare to controls including Mock, *gspd-1(RNAi)*, *idh-1* and *idh-2* mutant as well as *idh-1*(*RNAi*); *idh-2* and *idh-1*; *idh-2*(*RNAi*) double-deficient *C. elegans*, displayed shrinkage of body size (Fig. 1a). The *idh-1*;*gspd-1*(*RNAi*) double-deficient *C. elegans* also showed growth retardation (Supplementary Fig. S2) and slowed locomotion (Supplementary Fig. S3). The body size of *idh-1*;*gspd-1*(*RNAi*) double-deficient *C. elegans* was significantly decreased (*P* < 0.05) as determined by the perimeter and area measurements (Fig. 1b, c). Growth defect was not observed in *gspd-1*(*RNAi*) *C. elegans*. Neither the *idh-1* nor the *idh-2* mutation affected *C. elegans* growth. Likewise, *idh-2*;*gspd-1*(*RNAi*) double-deficient *C. elegans* had no reduction in body size.

**Fig. 2:**
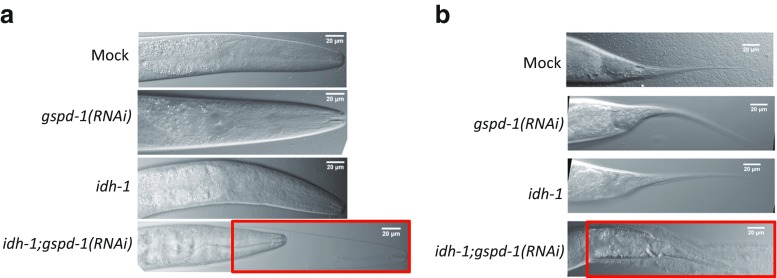
Molting defect of *idh-1*;*gspd-1*(*RNAi*) double-deficient *C. elegans* compared to mock and other controls. *Idh-1*;*gspd-1*(*RNAi*) double-deficient *C. elegans* showed a molting defect at the L4/Adult stage. Head (**a**) and tail (**b**) cuticle of *C. elegans* cultured at 20 °C for 54 h was photographed using a DIC microscope

The *idh-1*;*gspd-1*(*RNAi*) double-deficient *C. elegans* displayed an abnormal molting process, which was not observed in Mock, *gspd-1(RNAi)*, and *idh-1* mutant *C. elegans*. Such an abnormal molting process is demonstrated in Fig. 2a showing the molting defects at the head and Fig. 2b showing the molting defects at the tail. *Gspd-1* and *idh-1* suppression results in a disruption of normal molting indicating that *gspd-1* and *idh-1* are complementary to each other.

### Reduced NAS-37 protease expression in *idh-1*;*gspd-1*(*RNAi*) double-deficient *C*. *elegans*

**Fig. 3:**
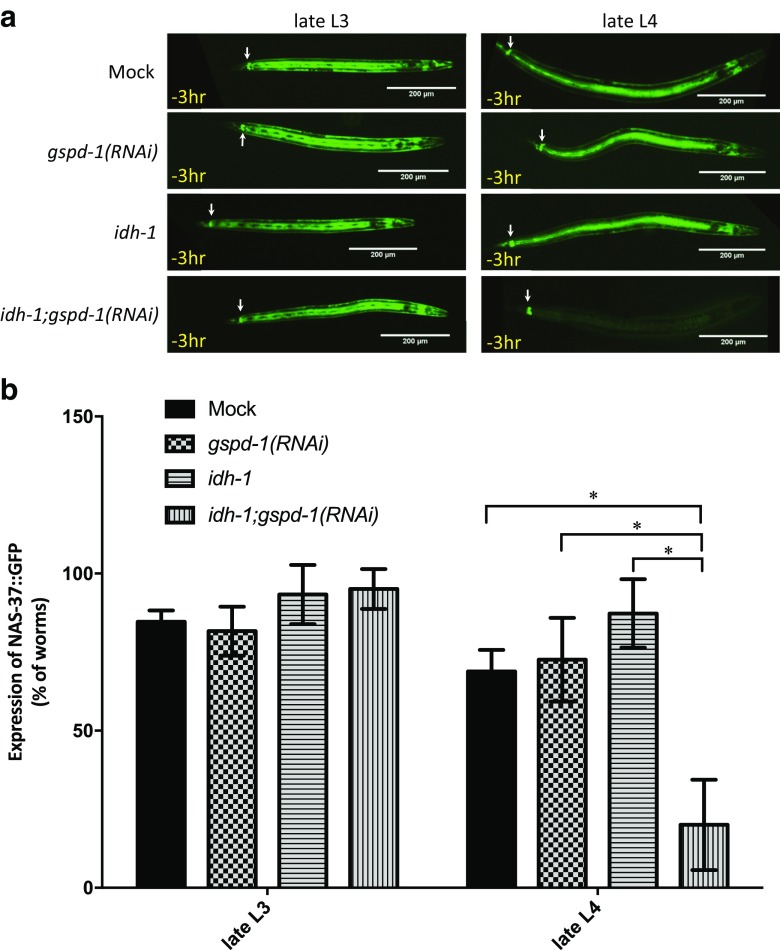
Decreased molting protein expression of *idh-1*;*gspd-1*(*RNAi*) double-deficient *C. elegans* compared to controls. *Idh-1*;*gspd-1*(*RNAi*) double-deficient *C. elegans* showed decreased molting protein NAS-37::GFP expression 3 h before the L4/adult molting. **a** NAS-37::GFP expression of *C. elegans* cultured at 25 °C for 28 h (3 h before L3/L4 molting) and 34 h (3 h before L4/adult molting) was photographed using a fluorescence microscope. The arrow points to the position of the rectal epithelial (REP) cells. **b** The ratio of NAS-37::GFP expression of L3 and L4 *C. elegans* was analyzed. (*n* > 60, *P < 0.05)

Molting is critical for transition between larval stages in nematodes. In *C. elegans*, three key steps, including apolysis (breaking the connection between old cuticle to the hypoderm), cuticle synthesis, and ecdysis (shedding the old cuticle when new cuticle is formed), must be precisely executed in order to grow and survive. Isolation of the mutations responsible for defective ecdysis identifies that *nas-37* is responsible for such a phenotype [29]. The *idh-1*;*gspd-1*(*RNAi*) double-deficient *C. elegans* (Fig. 2a) phenocopied the ecdysis mutants in which the cuticle cannot be shed. The *nas-37::gfp* fusion reporter strain of NAS-37 protease was used to determine whether or not the protein expression was affected during the molting process [29]. The expression level of NAS-37::GFP in all tested *C. elegans* was unaffected at late L3 (Fig. 3a). At late L4, the NAS-37::GFP signal of *idh-1*;*gspd-1*(*RNAi*) double-deficient *C. elegans* was reduced, compared with Mock, *gspd-1(RNAi)*, and *idh-1* mutant *C. elegans* (70% lower than that of Mock *C. elegans*, *P* < 0.05) (Fig. 3b). Consistently, reduced NAS-37::GFP expression level of *idh-1*;*gspd-1*(*RNAi*) double-deficient *C. elegans* at late L4 was found both at 25 °C (Fig. 3) and 20 °C (Supplementary Fig. S5). This indicates that sufficient NADPH derived from either GSPD-1 or IDH-1 or both is essential for NAS-37 protein expression to maintain normal molting at late L4 in *C. elegans*.

### Metabolomic amino acid analysis in *idh-1*;*gspd-1*(*RNAi*) double-deficient *C.* *elegans*

**Fig. 4:**
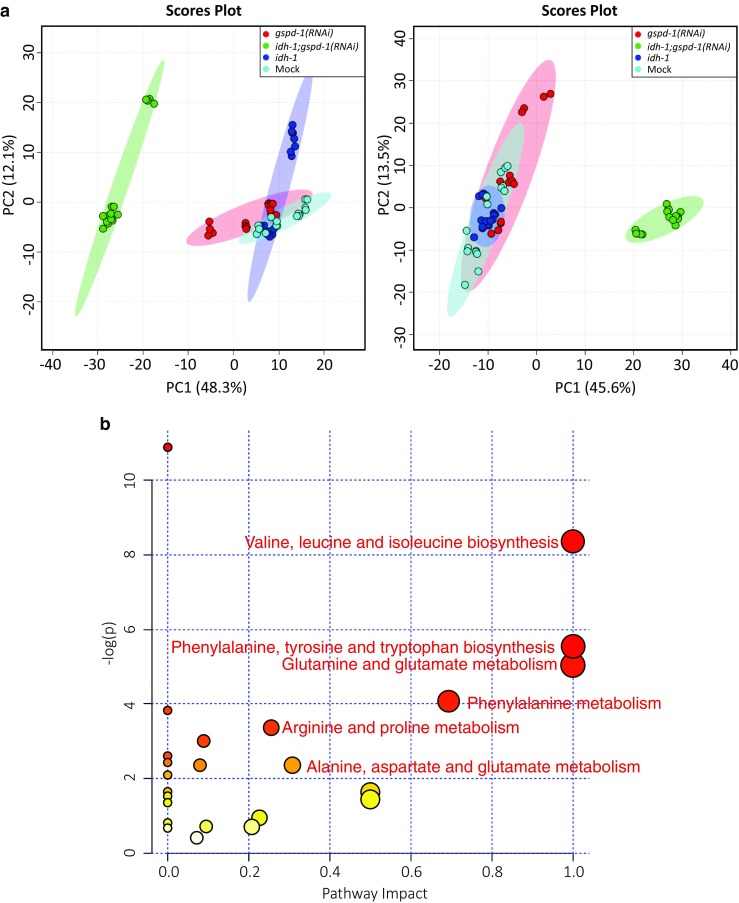
Distinct metabolic alterations of *idh-1*;*gspd-1*(*RNAi*) double-deficient *C. elegans*. **a** Data were subject to principal component analysis, and the score plots (left panel: ESI positive, right panel: ESI negative; Mock: cyan, *gspd-1* deficiency: red, *idh-1*: blue, *idh-1*;*gspd-1*(*RNAi*) double deficiency: green) were shown. Colored areas represented 95% confidence regions. **b** Pathway analysis of datasets indicated potential pathways that were significantly changed in *idh-1*;*gspd-1*(*RNAi*) double-deficient *C. elegans*. The global metabolomic view displays all anabolic and catabolic pathways that are ranked based on scores from pathway topology analysis (X-axis: pathway impact) and from pathway enrichment analysis (Y-axis: log(p))

Since decreased protein expression could be, in part, attributed to impaired amino acid metabolism, the metabolomics technique was employed to profile metabolomes of *idh-1*;*gspd-1*(*RNAi*) double-deficient *C. elegans* compared to controls. *Idh-1;gspd-1(RNAi)* double-deficient *C. elegans* exhibited distinct metabolic abnormalities as indicated by metabolomics analyses. The principal component analysis (PCA) model showed that while Mock control, *gspd-1(RNAi)*, and *idh-1*-deficient *C. elegans* were clustered together, *idh-1*;*gspd-1*(*RNAi*) double-deficient *C. elegans* was separated from the rest of the groups regardless of ESI positive or negative modes (Fig. 4a). The altered metabolites found in *idh-1*;*gspd-1*(*RNAi*) double-deficient *C. elegans* were selected in the ESI positive mode (90 metabolites out of 621 candidates) and in the ESI negative mode (34 metabolites out of 223 candidates). Fourteen metabolites of the ESI positive mode and ten metabolites of the ESI negative mode were identified by searching these selected metabolites against our metabolite database [30, 31]. Based on the pathway analysis, the metabolic pathways of several amino acids were found to be greatly affected. These amino acids included valine, leucine, isoleucine, phenylalanine, tyrosine and tryptophan biosynthesis, glutamine, glutamate, phenylalanine, arginine, and proline metabolism (Fig. 4b).

**Fig. 5:**
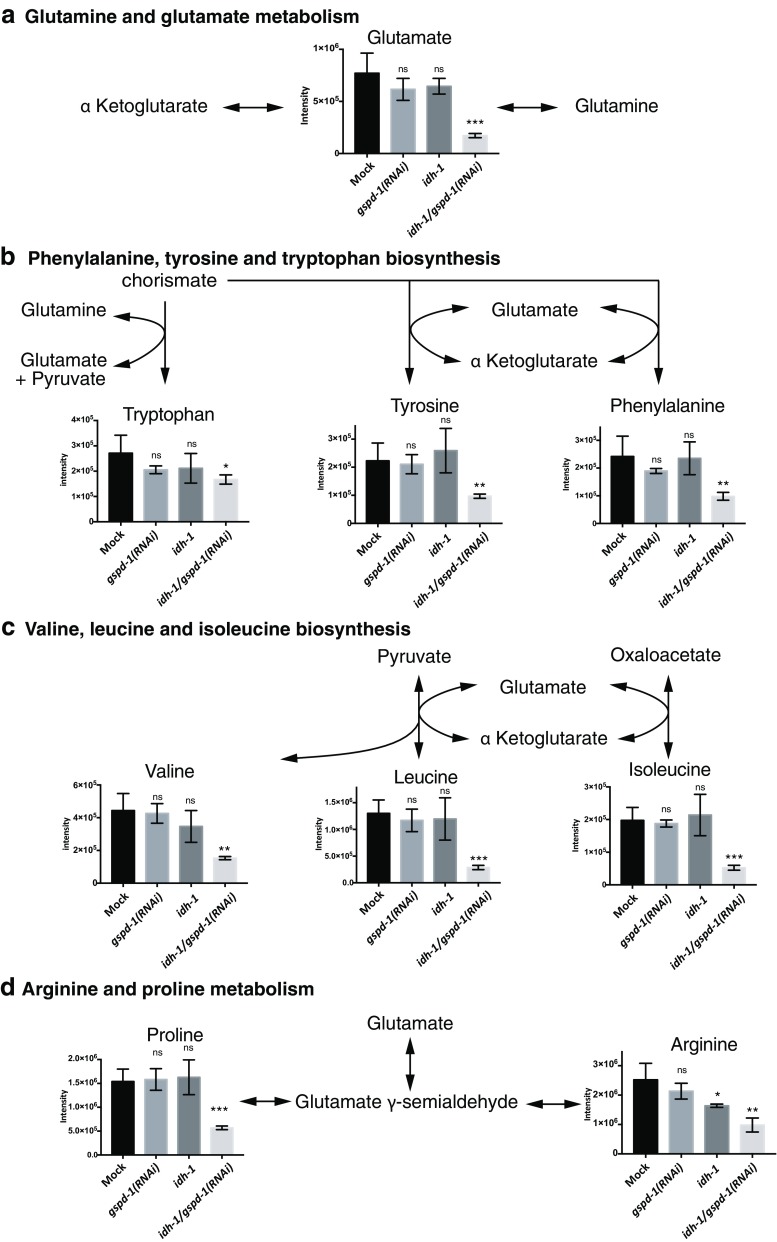
Altered amino acid metabolism in *idh-1*;*gspd-1*(*RNAi*) double-deficient *C. elegans*. **a** Glutamine and glutamate metabolism. **b** Phenylalanine metabolism. **c** Valine, leucine, and isoleucine biosynthesis. **d** Arginine and proline metabolism. All data were presented as the mean ± S.D.. and the statistical difference was analyzed by the two-tailed *t* test. (*P < 0.05; **P < 0.005; ***P < 0.001)

Quantitative analysis indicated that several amino acids from *idh-1*;*gspd-1*(*RNAi*) double-deficient *C. elegans* were significantly reduced compared with Mock (Fig. 5a–c). The glutamate level of *idh-1*;*gspd-1*(*RNAi*) double-deficient *C. elegans* decreased significantly to 22% of mock (*P* < 0.001) (Fig. 5a). The level of tryptophan was reduced to 63% in the *idh-1*;*gspd-1*(*RNAi*) double-deficient *C. elegans* compared with that of Mock (*P* < 0.05) (Fig. 5b), whereas the level of tryptophan of *gspd-1(RNAi)* or *idh-1* mutant *C. elegans* showed no difference compared with Mock. Phenylalanine and tyrosine levels were reduced in *idh-1*;*gspd-1*(*RNAi*) double-deficient *C. elegans* compared with Mock (41% and 34% of mock, respectively, *P* < 0.001), while they were unchanged in *idh-1* mutant *C. elegans*, and *gspd-1(RNAi)C. elegans* (Fig. 5b). The biosynthesis of valine, leucine, and isoleucine was reduced in *idh-1*;*gspd-1*(*RNAi*) double-deficient *C. elegans* compared with Mock (34% of mock, *P* < 0.005; 22% of mock, *P* < 0.001; 26% of mock, *P* < 0.001, respectively) (Fig. 5c). The level of arginine and proline metabolism was decreased in *idh-1*;*gspd-1*(*RNAi*) double-deficient mutant compared with Mock (39% of mock, *P* < 0.005 and 37% of mock, *P* < 0.001, respectively) (Fig. 5d).

## Discussion

Embryos derived from *C. elegans* fed with *Escherichia coli* expressing RNA-mediated interference (RNAi)-targeting *gspd-1* gene display developmental defects in their embryos, including hatching, membrane function, and eggshell structure [5, 6]. No defect in larval development is observed in the first generation of *gspd-1(RNAi)C. elegans* compared to control *C. elegans*. Perhaps, residual GSPD-1 activity and another NADPH-producing system can generate sufficient NADPH, in *gspd-1(RNAi)C. elegans*, to meet the basic requirement for larval growth until the reproduction period. It is not clear how IDH-1, another NADPH-producing system, can affect growth and development in GSPD-1 deficiency.

**Fig. 6:**
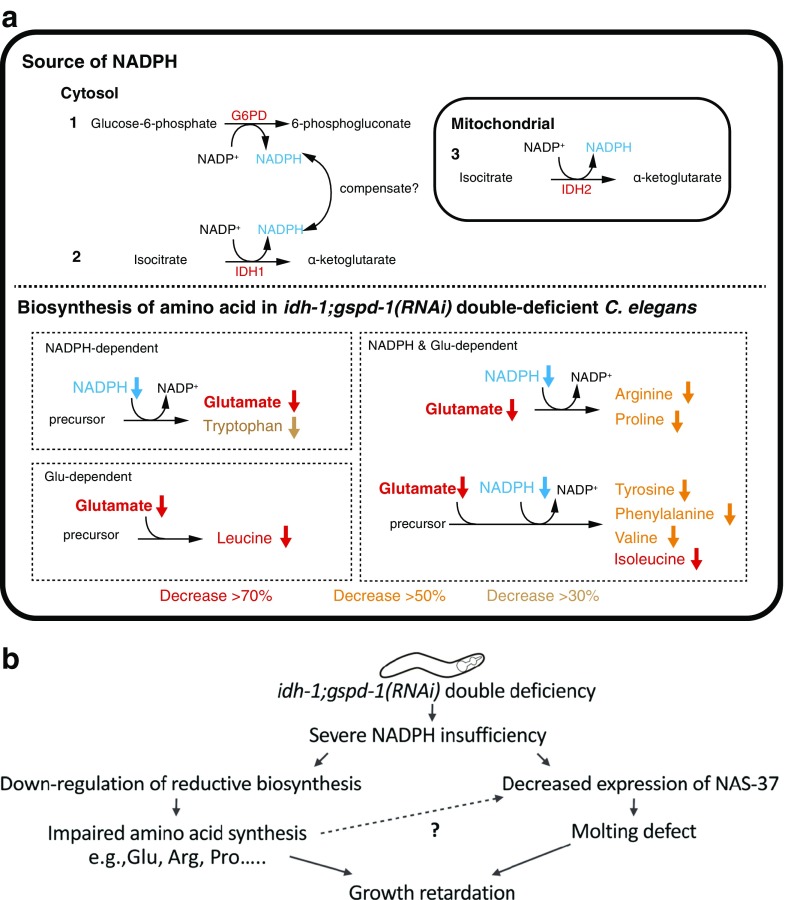
Proposed schemes for amino acid metabolism as well as for growth and development in *idh-1*;*gspd-1*(*RNAi*) double-deficient *C. elegans*. **a** Upper panel depicts three NADPH producing systems in *C. elegans*. Both GSPD-1 and IDH-1 are cytosolic enzymes that produce NADPH, whereas IDH-2 is the source of NADPH in mitochondria. Lower panel shows altered amino acid pathways in *idh-1*;*gspd-1*(*RNAi*) double-deficient *C. elegans*. These pathways are classified into three categories based on the requirement of NADPH or glutamate in amino acid synthesis. The decreased levels of amino acids are highlighted in red (> 70%), orange (> 50%), and beige (> 30%). **b** Hypothetical scheme of the physiological response in *idh-1*;*gspd-1*(*RNAi*) double-deficient *C. elegans*. The impaired amino acid synthesis may be due to the lack of NADPH for reductive biosynthesis. As a result, defective protein expression fails to support the growth of *C. elegans*. In addition, decreased expression of NAS-37 is linked to the molting defect, ultimately leading to retarded growth

In the current study, the small body size of *idh-1*;*gspd-1*(*RNAi*) double-deficient *C. elegans* is consistent with a previous report [32]. Similar findings are seen in yeast and nematode models lacking both G6PD and IDH [20, 32]. The distinct metabolomic profile of *idh-1*;*gspd-1*(*RNAi*) double-deficient *C. elegans* suggests that severe NADPH insufficiency causes a major disturbance in metabolism and is linked to its defective phenotypes, including reduced body size and impaired molting in *C. elegans*. It is speculated that the compensation of the complementary NADPH-producing systems supports reductive biosynthesis and provides sufficient reducing power to meet the need of cells (Fig. 6a).

Essential amino acids, including arginine, histidine, isoleucine, leucine, lysine, methionine, phenylalanine, threonine, tryptophan, and valine, are required to support the growth of *C. elegans* [21, 33]. The global metabolomic analysis revealed that amino acid biosynthesis and metabolism were significantly affected by the diminution of both GSPD-1 and IDH-1 in *C. elegans*. Since glutamate dehydrogenase requires NADPH to synthesize glutamate from α-ketoglutarate and NH_4_^+^, the level of glutamate was decreased in *idh-1*;*gspd-1*(*RNAi*) double-deficient *C. elegans*. Glutamate is a precursor of the non-essential amino acids, namely proline and arginine [21], which were reduced in *idh-1*;*gspd-1*(*RNAi*) deficient *C. elegans*. The essential branched chain amino acids, such as valine, leucine, and isoleucine, were decreased in *idh-1*;*gspd-1*(*RNAi*) double-deficient *C. elegans* due to the requirement of NADPH for the biosynthesis of these amino acids. In addition, the levels of tryptophan and phenylalanine were decreased in *idh-1*;*gspd-1*(*RNAi*) double-deficient *C. elegans.* The fact that decreased amino acid pool found in *idh-1*;*gspd-1*(*RNAi*) double-deficient *C. elegans* raises a question whether supplementation with amino acids rescues the growth defect. Preliminary study to address this issue is already being undertaken by our group; however, more detailed works are needed (Supplementary text). Taken together, decreased amino acids found in *idh-1*;*gspd-1*(*RNAi*) double-deficient *C. elegans* reiterate the importance of the NADPH supply in reductive biosynthesis during organismal development (Fig. 6a).

The decrease in amino acids which requires NADPH in their biosynthetic pathways suggests that severe insufficiency of NADPH may affect protein synthesis as illustrated by the impaired molting process in *idh-1*;*gspd-1*(*RNAi*) double-deficient *C. elegans* model. *C. elegans* molting is a tissue remodeling process, which requires the activity of proteases to degrade old cuticle proteins. The status of proteases can lead to molting defects. A decrease in the level of NAS-37 expression in *idh-1*;*gspd-1*(*RNAi*) double-deficient *C. elegans* during the larval stage suggests that the protease is affected by NADPH insufficiency. *Nas-37* is expressed in the hypodermis prior to ecdysis at each larval stage in *C. elegans* [29]. The gene product of *nas-37*, an Astacin-class metalloprotease, accumulates in the anterior cuticle and is secreted to degrade the old cuticle after ecdysis. A scheme is outlined that describe the effects of *idh-1*;*gspd-1*(*RNAi*)1 double deficiency in *C. elegans* (Fig. 6b). NADPH is an important fuel to drive the development machinery. Decreased amino acid synthesis is a main metabolic alteration leading to growth retardation in *idh-1*;*gspd-1*(*RNAi*) double-deficient *C. elegans*. In addition, decreased NAS-37 expression and the molting defect are likely to cause growth retardation through the unshed cuticle restricting the growth of *idh-1*;*gspd-1*(*RNAi*) double-deficient *C. elegans*. Solid evidence to prove a causal relationship between NAS-37 expression and impaired amino acid synthesis awaits further investigation.

Currently, there is little information of the relationship between NADPH and NAS-37 or molting in *C. elegans*. A few studies indicate that a collagen-modifying enzyme, known as NADPH dual oxidase or Bli-3, catalyzes the crosslinking of cuticular collagens [34, 35]. In the current study, however, the blister phenotype of *bli-3* mutant has not been observed in *idh-1*;*gspd-1*(*RNAi*) double-deficient *C. elegans*. Hence, another yet to be identified target(s) may be modulated by NADPH depletion leading to growth defects in *C. elegans.* Abnormal molting in *idh-1*;*gspd-1*(*RNAi*) double-deficient *C. elegans* could be, in part, attributed to abnormal fatty acid synthesis because genes of fatty acid synthesis participate in the molting process in *C. elegans* [36]. Knockdown of *fasn-1* and *pod-2* by RNAi downregulates the protein expression of NAS-37. Since NADPH is required for de novo fatty acid synthesis, it is possible that GSPD-1 and IDH-1 double-deficiency-derived NADPH insufficiency disrupts fatty acid biosynthesis leading to NAS-37 inactivation and the molting defect.

G6PD status is associated with many human diseases, including hemolytic disorders, cardiovascular diseases, and diabetes [2]. Although the relationship between G6PD and cancer is unclear, G6PD is involved in transformation and angiogenesis [37]. Overexpression and modification of G6PD promotes tumor growth and leads to a poor clinical outcome [38–43], while suppression of G6PD inhibits cancer development [42, 44–46]. The notion that G6PD favors rapid cell proliferation is consistent with the finding that inactivation of G6PD causes embryonic lethality [5, 6]. In short, the current study provides evidence for the involvement of G6PD and IDH1 in cellular as well as organismal growth and development. Similar to G6PD, IDHs play a diverse role in pathophysiology [47–49].

G6PD-derived NADPH is implicated in maintaining redox homeostasis and reductive biosynthesis [3]. Metabolomic studies have demonstrated that G6PD participates in the rapid response of metabolic rerouting to counteract oxidative stress [30, 50]. Lipidomic analysis of embryos derived from GSPD-1-deficient *C. elegans* exhibit higher levels of lipid peroxidation [6]. Increased expression of G6PD in the transgenic mice model shows the enhanced NADPH production and reduces oxidative damage [51]. G6PD transgenic mice exhibit improved protection against an age-related functional decline and an extended health span in females. The metabolomic approach employed in the current study provides additional information concerning the complementary role of GSPD-1 and IDH-1 in maintaining cellular redox homeostasis and “redox-regulated biosynthesis.”

## Materials and methods

### Nematode culture and RNAi silencing

N2 (wild type), *idh-1*(*ok2832*), and *idh-2*(*ok3184*) mutants were acquired from *Caenorhabditis* Genetics Center (University of Minnesota, Minneapolis, USA). The NAS-37 reporter strain EG3198 was a gift from Prof. Erik Jorgensen (University of Utah, Salt Lake City, USA). The strains were maintained at 20 °C on Nematode Growth Medium (NGM) agar plate seeded with live *E. coli OP50* bacterial lawn based on standard protocols [52]. The *gspd-1*-RNAi silencing experiment was performed as described previously [5, 6]. The *idh-1* and *gspd-1* double-deficient strain was created by using *gspd-1* RNAi in *idh-1*(*ok2832*) background. The *idh-2* and *gspd-1* double-deficient strain was created by using *gspd-1* RNAi in *idh-2*(*ok3184*) background. The RNAi strains, using live *E. coli HT115*(*DE3*) as a food source, were maintained at 20 °C on NGM agar plate supplemented with 1 mM IPTG.

### Phenotype assays

For the molting assay, synchronized L1 grown at 20 °C for 48 h was picked and mounted on a 2% agarose pad on a glass slide followed by anesthetizing with 0.2% levamisole, and a cover slide was placed on the agarose pad. DIC and fluorescent images were taken by using an epifluorescence microscope (Leica, Wetzlar, Germany). For the body size and locomotion assays, synchronized L1 was cultivated on NGM plates at 20 °C for 72 h. Images and video clips were taken by using a dissecting microscope (Nikon, Japan) with a MoticCam X CMOS camera (Motic, Xiamen, China) followed by image analysis (Metamorph 6.1r0; Molecular Device, CA, USA).

### Metabolomic analysis

The synchronized L1 worms were cultured at 20 °C until adulthood. Four biological replicates of adult worms were washed off NGM plates by ultra-water. The samples were washed twice by M9 buffer and were centrifuged at 2500 rpm for 1 min. The samples were suspended with 80% methanol and transferred to a homogenization tube pre-filled with 1.0-mm diameter Zirconia beads (Biospec, Bartlesville, OK, USA) and were homogenized in a Precellys24 homogenizer coupled with a Cryolys Cooling System (Bertin Instrument, Rockville, MD, USA). The homogenization was set at two cycles of 6500 rpm for 30 s with an interval of 5 s. Subsequently, the samples were centrifuged at 12000 rpm for 15 min at 4 °C. The supernatant of each sample was transferred to a separate glass tube. The homogenization tube was refilled with 1 ml of 80% methanol followed by additional homogenization and centrifugation to recover residual samples. The supernatant was transferred to the previous glass tube and air dried under nitrogen flow in a nitrogen evaporator (Taitec, Koshigaya-shi, Saitama-ken, Japan) and stored at − 80 °C. The pellet was dissolved in 1 ml of 0.1 N NaOH at 65 °C for 30 min to determine the protein concentration (Bradford assay).

Prior to metabolomic analysis, the sample was dissolved in 300 μl LC-MS Chromasolv water (Fluka) containing 0.1% formic acid. The tube was vortexed for 30 s and repeated four times. The mixture was transferred to an eppendorf tube and centrifuged at 12000 rpm for 30 min at 4 °C. The supernatant was then transferred to an HPLC vial for liquid chromatography and mass spectrometry analysis. For LC-MS analysis, mass spectrometry analysis was carried out using an Agilent 1200 rapid resolution liquid chromatography system coupled with an Agilent 6510 Q-TOF MS system (Agilent Technologies, CA, USA), which is equipped with an electrospray ionization source. Chromatographic separation was performed on an Acquity UPLC HSST3 reversed phase C18 column (particle size of 1.8 μm, 2.1 mm × 150 mm) (Waters, Milford, USA). Column temperature was maintained at 40 °C and the flow rate was 0.25 ml/min. For metabolite profiling, the mobile phase consisted of 0.1% formic acid (solvent A) and 0.1% formic acid/acetonitrile (solvent B). The mobile phase condition was listed below: solvent A, 2 min; gradient from 0 to 40% solvent B, 4 min; 40% solvent B, 2 min; gradient from 40 to 98% solvent B, 2 min; 98% solvent B, 6 min; gradient from 98 to 0% solvent B, 2 min.

The samples were subjected to RRLC-ESI-TOF-MS. Mass spectrometric analysis was performed in the ESI positive and ESI negative modes. The pressure of the nitrogen nebulizer was set at 30 psi and the nitrogen drying gas was set at 350 °C with a flow rate of 10 l/min. The skimmer and capillary voltages were set at 65 V and 4000 V, respectively. Data were obtained over the range from m/z 50 to m/z 1000 at a rate of 1 scan per second. Data were collected in the profile mode using Agilent MassHunter Workstation Data acquisition software. For processing data, individual components, or called molecular features, in the sample were identified using the Molecular Feature Extraction (MFE) algorithm of MassHunter software. Upon processing, the raw data generated time-aligned ion features (isotopes, adducts, and dimers), the monoisotopic neutral mass, retention time, and ion abundance for each molecular feature. An Agilent GeneSpring-MS (Agilent Technologies, CA, USA) was used to visualize datasets in numerical data matrices (metabolite concentrations). MetaboAnalyst 3.0 was used for analysis and visualization of MS data sets in data matrices and principal components analysis (PCA) diagrams as well as multivariate data analysis and data representation, such as a volcano plot. PCA was employed for clustering and correlation analyses. Relative concentrations of metabolites were compared by ANOVA with a Tukey HSD correction. Accurate masses of features showing significant differences between control and test groups were searched against our in-house and public metabolite databases, including HMDB (http://www.hmdb.ca), METLIN (http://metlin.scripps.edu/index.php), and KEGG (http://www.genome.jp/kegg/).

### Statistical analysis

Where applicable, all data were shown as the mean ± S.D. The statistical difference was analyzed by the two-tailed *t* test. All statistical tests were conducted using the GraphPad Prism 6.0 (San Diego, CA, USA). Values of *P* < 0.05 were considered statistically significant.

## Electronic supplementary material

ESM 1 (DOCX 17 kb)

Fig. S1 Comparison of G6PD activity among Mock, *idh-1*, *idh-2*, *gspd-1(RNAi)*, *idh-1;gspd-1(RNAi)* and *idh-2;gspd-1(RNAi) C. elegans*. G6PD activity remained unchanged in Mock or in *idh-1* and *idh-2* mutant *C. elegans*, while G6PD activity was reduced in *gspd-1(RNAi)*, *idh-1;gspd-1(RNAi)* and *idh-2;gspd-1(RNAi) C. elegans*. Protein samples were extracted from adult *C. elegans* followed by G6PD activity analysis. (*N* = 4, *: *P* < 0.05) (PNG 48 kb)

High Resolution Image (EPS 149 kb)

Fig. S2 Decreased body size of *idh-1;gspd-1(RNAi)* double-deficient *C. elegans* compared to controls. The size of *idh-1;gspd-1(RNAi)* double-deficient *C. elegans* was decreased as compared to other *C. elegans* strains at day 3(a), day 5(b) or day 8(c). *C. elegans* was examined by image analysis software by using a dissecting microscope. The black scale bar represented 0.5 mm. (PNG 860 kb)

High Resolution Image (EPS 4665 kb)

Fig. S3 Stationary locomotion of *idh-1;gspd-1(RNAi)* double-deficient *C. elegans* compared to Mock, *idh-1*, *gspd-1(RNAi)*. Locomotion tracks of *idh-1;gspd-1(RNAi)* double-deficient *C. elegans* were mostly stationary while other strains moved ahead. The red undulating line represented the locomotion track of adult *C. elegans* during 20 s of recording. The black scale bar represented 1 mm. (1128 kb)

High Resolution Image (EPS 3237 kb)

Fig. S4 Distinct metabolic alterations of *idh-1;gspd-1(RNAi)* double-deficient *C. elegans*. Data were subject to principal component analysis, and the score plots (left panel: ESI positive, right panel: ESI negative; Mock: cyan, *gspd-1(RNAi)* deficiency: red, *idh-1*: blue, *idh-1;gspd-1(RNAi)* double deficiency: green, QC (Quality control): magenta) were shown. Colored areas represented 95% confidence regions. (PNG 309 kb)

High Resolution Image (EPS 2952 kb)

Fig. S5 Decreased molting protein expression of *idh-1;gspd-1(RNAi)* double-deficient *C. elegans* compared to controls at 20 °C. *idh-1;gspd-1(RNAi)* double-deficient *C. elegans* showed decreased molting protein NAS-37::GFP expression before the L4/adult molting. The ratio of NAS-37::GFP expression of L4 *C. elegans* was analyzed. (*n* > 60, *: *P* < 0.05) (PNG 44 kb)

High Resolution Image (EPS 183 kb)
