# Supplementary material for: IDH-1 deficiency induces growth defects and metabolic alterations in GSPD-1-deficient Caenorhabditis elegans
Source: J Mol Med (Berl). 2019 Jan 19;97(3):385–96. doi: 10.1007/s00109-018-01740-2 (PMC6394583; doi:10.1007/s00109-018-01740-2)
Supplement: Supplementary file 1 — (DOCX 17 kb) [file 109_2018_1740_MOESM1_ESM.docx]

**Supplementary text**

In order to address whether supplementation with amino acids rescues the growth defect of *idh-1;gspd-1(RNAi)* double-deficient *C. elegans*, we performed amino acid supplementation experiment based on a previous protocol [1]. In brief, *idh-1;gspd-1(RNAi)* double-deficient *C. elegans* was cultured in liquid medium supplementing amino acids which were reduced indicated by the metabolomic analysis. A mixture of nine amino acids (indicated in Fig. 5) or individual amino acid was supplemented. However, neither of the approach significantly reversed the growth defects. It is assumed that due to the complex amino acid biosynthesis and metabolism *in vivo*, the dose and delivery of exogenous amino acid may be suboptimal to rescue the defective growth caused by GSPD-1 and IDH-1 deficiency. It is anticipated that alternative pathway yet to be identified may link to altered amino acid metabolism and the defective growth.

**Supplementary reference**

1. Edwards C, Canfield J, Copes N, Brito A, Rehan M, Lipps D, Brunquell J, Westerheide SD, Bradshaw PC (2015) Mechanisms of amino acid-mediated lifespan extension in Caenorhabditis elegans. BMC Genet 16: 8. DOI 10.1186/s12863-015-0167-2
