# Supplementary figures and images for: IDH-1 deficiency induces growth defects and metabolic alterations in GSPD-1-deficient Caenorhabditis elegans
Source: J Mol Med (Berl). 2019 Jan 19;97(3):385–96. doi: 10.1007/s00109-018-01740-2 (PMC6394583; doi:10.1007/s00109-018-01740-2)

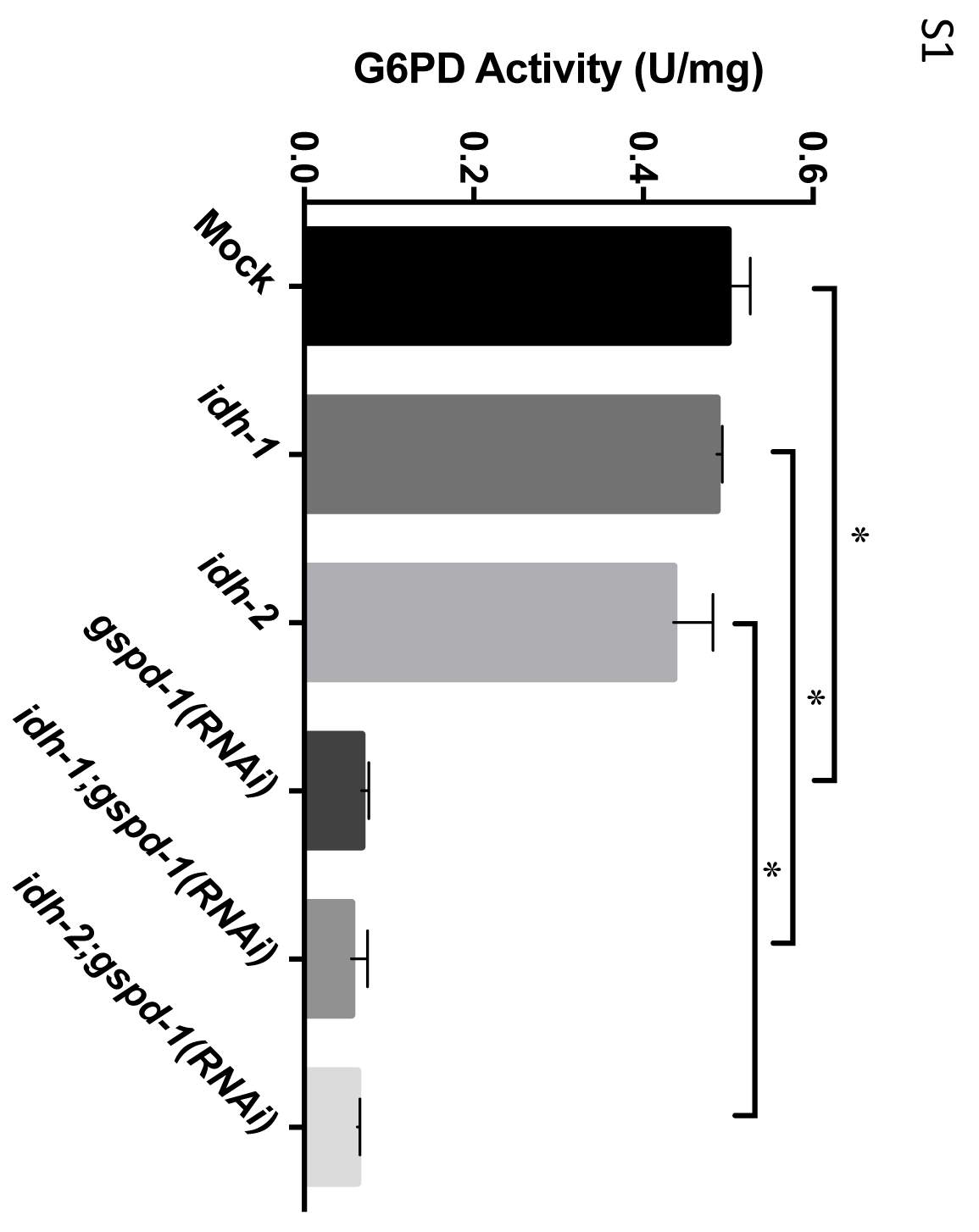

Supplement: Supplementary file 2 — Comparison of G6PD activity among Mock, idh-1, idh-2, gspd-1(RNAi), idh-1;gspd-1(RNAi) and idh-2;gspd-1(RNAi) C. elegans. G6PD activity remained unchanged in Mock or in idh-1 and idh-2 mutant C. elegans, while G6PD activity was reduced in gspd-1(RNAi), idh-1;gspd-1(RNAi) and idh-2;gspd-1(RNAi) C. elegans. Protein samples were extracted from adult C. elegans followed by G6PD activity analysis. (N = 4, *: P < 0.05) (PNG 48 kb) [file 109_2018_1740_Fig7_ESM.png]

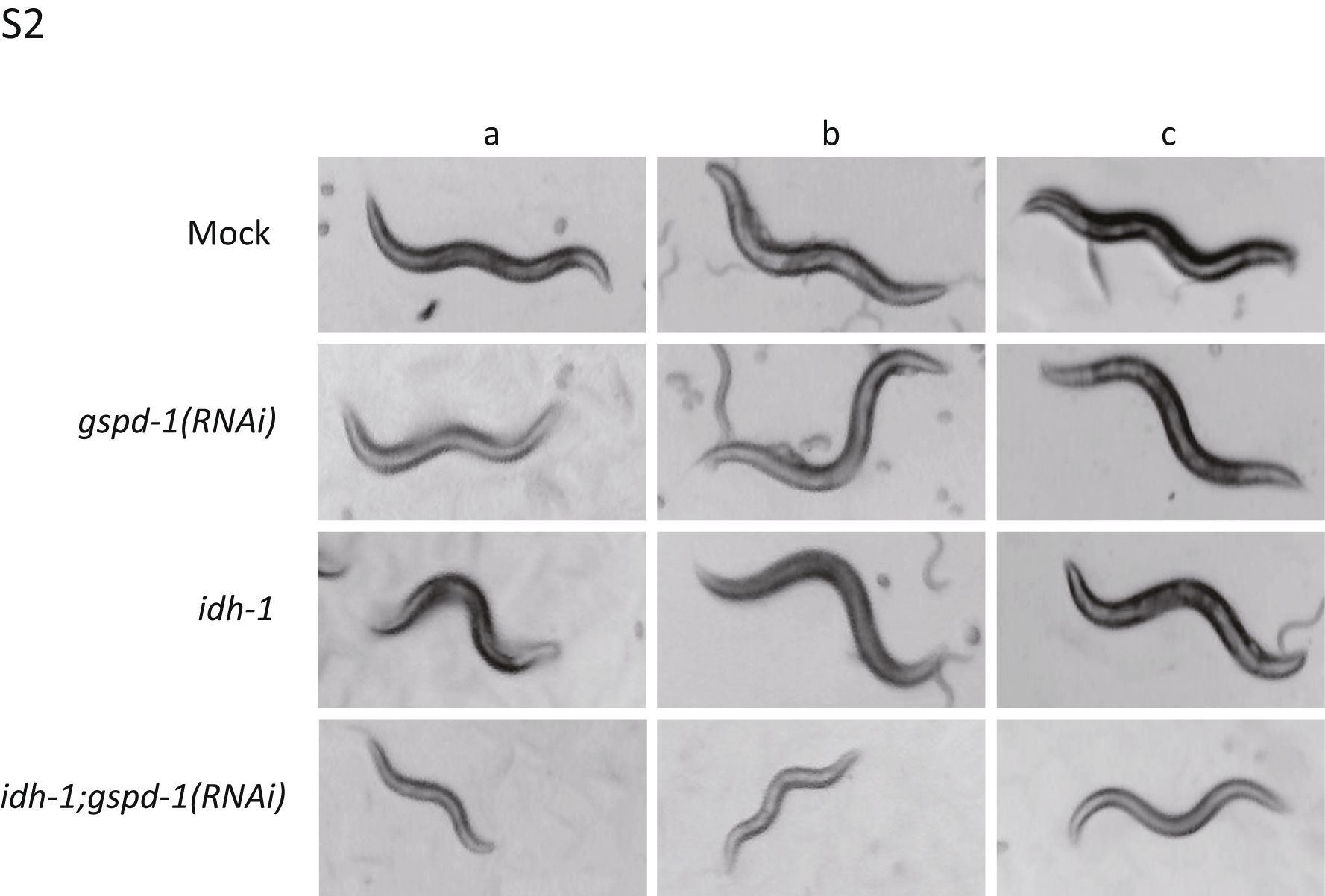

Supplement: Supplementary file 4 — Decreased body size of idh-1;gspd-1(RNAi) double-deficient C. elegans compared to controls. The size of idh-1;gspd-1(RNAi) double-deficient C. elegans was decreased as compared to other C. elegans strains at day 3(a), day 5(b) or day 8(c). C. elegans was examined by image analysis software by using a dissecting microscope. The black scale bar represented 0.5 mm. (PNG 860 kb) [file 109_2018_1740_Fig8_ESM.png]

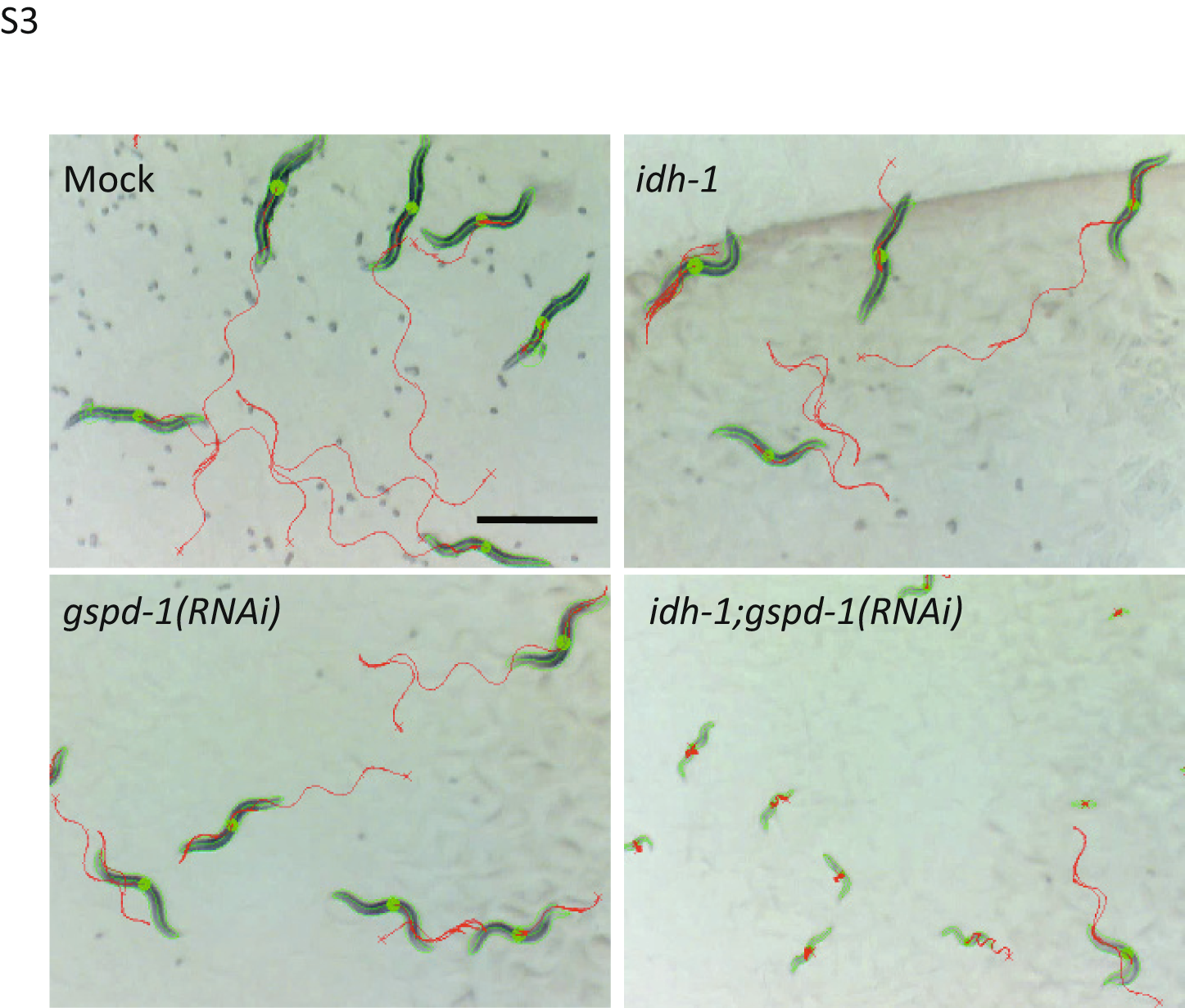

Supplement: Supplementary file 6 — Stationary locomotion of idh-1;gspd-1(RNAi) double-deficient C. elegans compared to Mock, idh-1, gspd-1(RNAi). Locomotion tracks of idh-1;gspd-1(RNAi) double-deficient C. elegans were mostly stationary while other strains moved ahead. The red undulating line represented the locomotion track of adult C. elegans during 20 s of recording. The black scale bar represented 1 mm. (1128 kb) [file 109_2018_1740_Fig9_ESM.png]

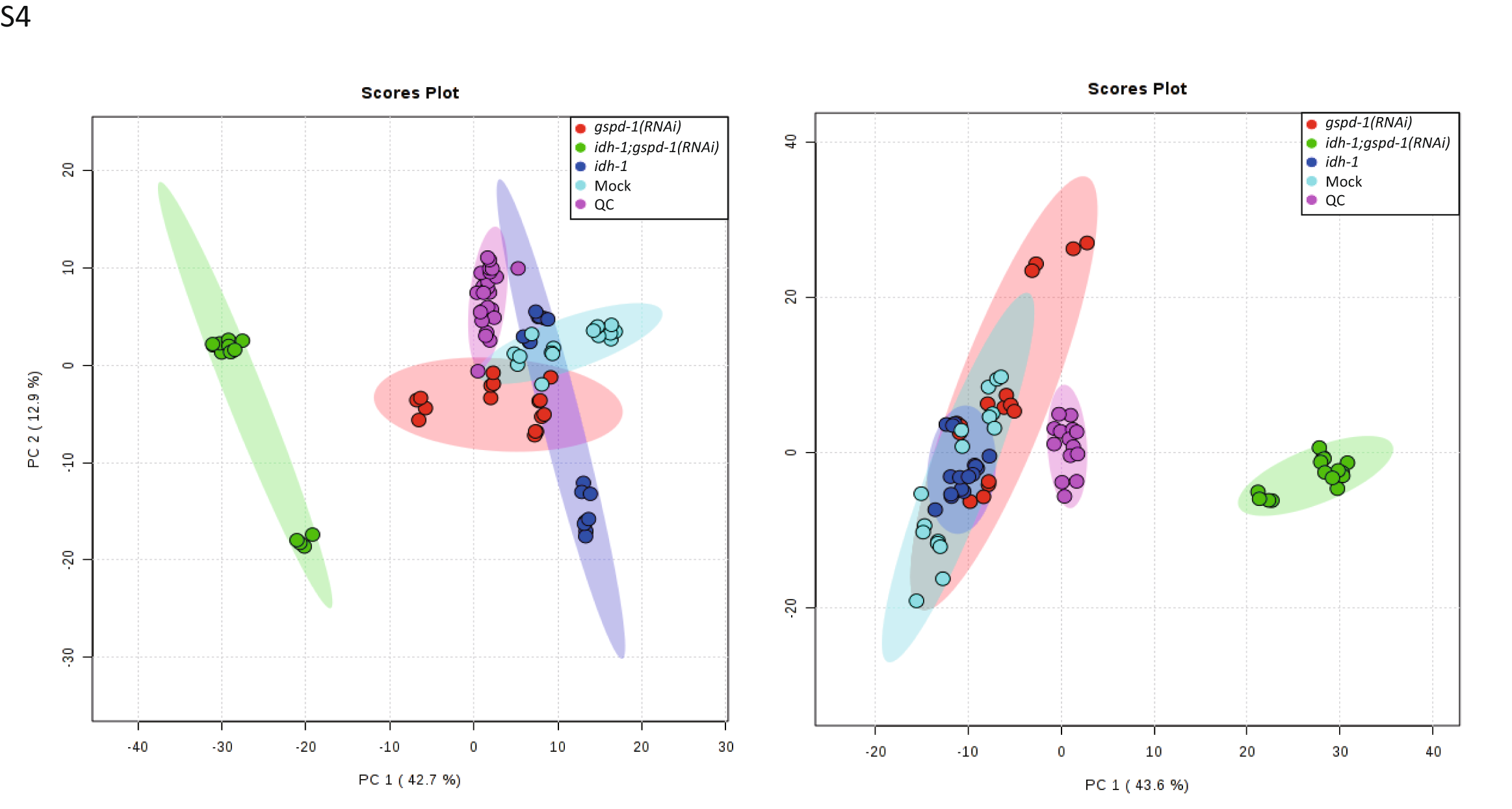

Supplement: Supplementary file 8 — Distinct metabolic alterations of idh-1;gspd-1(RNAi) double-deficient C. elegans. Data were subject to principal component analysis, and the score plots (left panel: ESI positive, right panel: ESI negative; Mock: cyan, gspd-1(RNAi) deficiency: red, idh-1: blue, idh-1;gspd-1(RNAi) double deficiency: green, QC (Quality control): magenta) were shown. Colored areas represented 95% confidence regions. (PNG 309 kb) [file 109_2018_1740_Fig10_ESM.png]

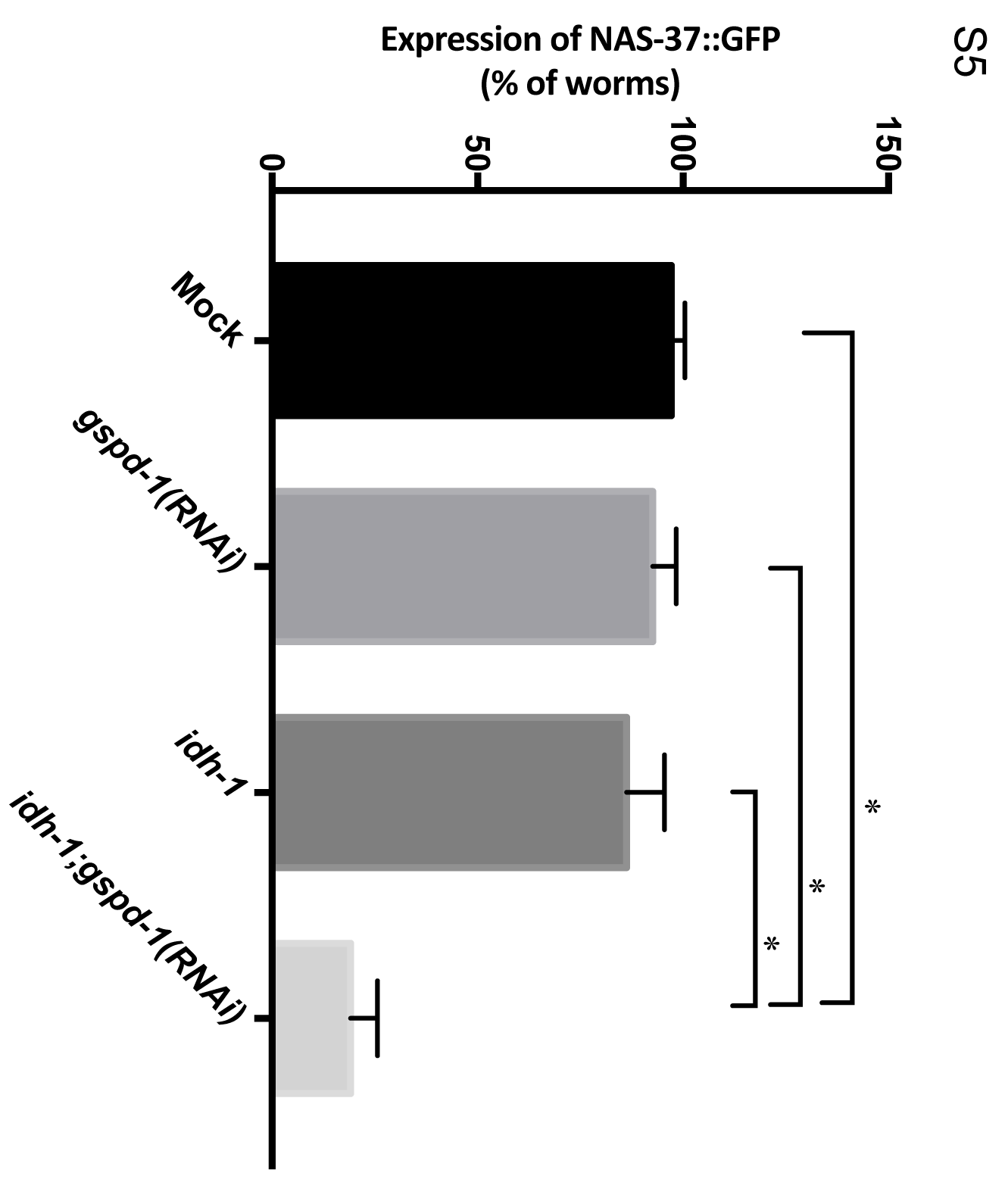

Supplement: Supplementary file 10 — Decreased molting protein expression of idh-1;gspd-1(RNAi) double-deficient C. elegans compared to controls at 20 °C. idh-1;gspd-1(RNAi) double-deficient C. elegans showed decreased molting protein NAS-37::GFP expression before the L4/adult molting. The ratio of NAS-37::GFP expression of L4 C. elegans was analyzed. (n > 60, *: P < 0.05) (PNG 44 kb) [file 109_2018_1740_Fig11_ESM.png]
